# Robust Bayesian meta‐analysis: Model‐averaging across complementary publication bias adjustment methods

**DOI:** 10.1002/jrsm.1594

**Published:** 2022-08-07

**Authors:** František Bartoš, Maximilian Maier, Eric‐Jan Wagenmakers, Hristos Doucouliagos, T. D. Stanley

**Affiliations:** ^1^ Department of Psychological Methods University of Amsterdam Amsterdam The Netherlands; ^2^ Institute of Computer Science Czech Academy of Sciences Prague Czech Republic; ^3^ Department of Experimental Psychology University College London London England UK; ^4^ Deakin Laboratory for the Meta‐Analysis of Research (DeLMAR) Deakin University Melbourne Australia; ^5^ Department of Economics Deakin University Melbourne Australia

**Keywords:** Bayesian model‐averaging, meta‐analysis, PET‐PEESE, publication bias, selection models

## Abstract

Publication bias is a ubiquitous threat to the validity of meta‐analysis and the accumulation of scientific evidence. In order to estimate and counteract the impact of publication bias, multiple methods have been developed; however, recent simulation studies have shown the methods' performance to depend on the true data generating process, and no method consistently outperforms the others across a wide range of conditions. Unfortunately, when different methods lead to contradicting conclusions, researchers can choose those methods that lead to a desired outcome. To avoid the condition‐dependent, all‐or‐none choice between competing methods and conflicting results, we extend robust Bayesian meta‐analysis and model‐average across two prominent approaches of adjusting for publication bias: (1) selection models of *p*‐values and (2) models adjusting for small‐study effects. The resulting model ensemble weights the estimates and the evidence for the absence/presence of the effect from the competing approaches with the support they receive from the data. Applications, simulations, and comparisons to preregistered, multi‐lab replications demonstrate the benefits of Bayesian model‐averaging of complementary publication bias adjustment methods.

## 1 INTRODUCTION

Meta‐analysis is essential to cumulative science.^1^ However, a common concern to meta‐analysis is the overestimation of effect size due to publication bias, the preferential publishing of statistically significant studies.^2^, ^3^, ^4^, ^5^, ^6^ In addition, this effect size exaggeration can be further increased by questionable research practices, that is, researchers' tendency to manipulate their data in a way that increases the effect size and the evidence for an effect.^7^, ^8^ Indeed, descriptive surveys find that both problems are remarkably common. For example, John and colleagues^9^ estimate that about 78% of researchers failed to disclose all dependent measures and around 36% stopped data collection after achieving a significant result* (but see Fiedler & Schwarz^10^ who argued that the survey by John and colleagues^9^ overestimated QRPs prevalence).

The results of the publication bias and questionable research practices are often viewed as a missing data problem; where some studies are missing from the published research record because they did not reach a statistical significance criterion while other estimates are observed after being “massaged” by researchers. Unfortunately, a perfect solution to the problem of missing data is impossible since we cannot know the unreported results nor the precise mechanism of omission. Multiple methods have been offered to adjust for likely publication bias from observable patterns contained in the reported research record.^11^, ^12^, ^13^, ^14^, ^15^, ^16^, ^17^, ^18^, ^19^, ^20^, ^21^, ^22^, ^23^, ^24^ All of these methods have been shown to thrive under different assumptions and simulation designs.^13^, ^25^, ^26^, ^27^

Because different methods can lead to different conclusions, some meta‐analysts suggest that we should not search for the “best” bias‐adjusted effect size estimate. Instead, they suggest that multitude bias‐adjusted effect size estimates should be offered as a sensitivity analysis for the original unadjusted value.^28^, ^29^, ^30^ The new method proposed here is another useful tool to accommodate publication bias, and researchers are free to supplement it with other methods.

Researchers interested in obtaining better bias‐adjusted effect size estimates or selecting the most suitable set of methods for sensitivity analysis increasingly emphasize the importance of selecting an appropriate estimator conditional on the situation at hand. For instance, Hong & Reed^27^ argue:

What is missing is something akin to a flow‐chart that would map observable characteristics to experimental results which the meta‐analyst could then use to select the best estimator for their situation. (p. 22)

And Carter and colleagues^25^ write:

Therefore, we recommend that meta‐analysts in psychology focus on sensitivity analyses—that is, report on a variety of methods, consider the conditions under which these methods fail (as indicated by simulation studies such as ours), and then report how conclusions might change depending on which conditions are most plausible. (p. 115)

In practice, researchers seldom have knowledge about the data‐generating process nor do they have sufficient information to choose with confidence among the wide variety of proposed methods that aim to adjust for publication bias. Furthermore, this wide range of proposed methods often leads to contradictory conclusions.^25^ The combination of uncertainty about the data‐generating process and the presence of conflicting conclusions can create a “breeding ground” for confirmation bias^31^: researchers may unintentionally select those methods that support the desired outcome. This freedom to choose can greatly inflate the rate of false positives, which can be a serious problem for conventional meta‐analysis methods.

An alternative approach is to integrate the different approaches, explicitly, and let the data determine the contribution of each model based on its relative predictive accuracy for the observed data. To implement this approach we extend the robust Bayesian meta‐analysis (RoBMA) framework outlined in Maier and colleagues.^13^ The original RoBMA framework included selection models (operating on *p*‐values) that have been shown to work well even under high heterogeneity^25^, ^32^ (see also Guan and colleagues for earlier work^33^). The extended RoBMA framework also includes PET‐PEESE, a method that adjusts for small‐study effects by modeling the relationship between the effect sizes and standard errors.^16^ PET‐PEESE generally has low bias and performs well in applications.^25^, ^34^ By including both *p*‐value selection models as well as PET‐PEESE, the extended version of RoBMA can apply both models simultaneously and optimally, relative to the observed research record.

Below we first provide a brief introduction to the RoBMA framework. We use an example on precognition^35^ to illustrate both the general model‐averaging methodology, RoBMA‐PSMA (PSMA: publication selection model‐averaging), and the way RoBMA‐PSMA combines multiple weight functions including PET‐PEESE. Second, we evaluate RoBMA‐PSMA on comparisons with findings from preregistered multi‐lab replications,^34^ and across more than a thousand simulation environments employed by four different simulation studies.^27^

## 2 ROBUST BAYESIAN META‐ANALYSIS: GENERAL BACKGROUND

Because the true data generating process is unknown (effect present vs. effect absent; fixed‐effect vs. random‐effects; no publication bias vs. publication bias; and how publication bias expresses itself), many different models can be specified. RoBMA‐PSMA accepts this multitude of models and uses Bayesian model‐averaging to combine the estimates from individual models based on how well each model predicts the data.^36^, ^37^, ^38^ Consequently, the posterior plausibility for each individual model determines its contribution to the model‐averaged posterior distributions.^13^, ^39^, ^40^

In this section, we provide a brief overview of Bayesian model‐averaging, the work horse of RoBMA (for an in‐depth treatment see^38^, ^40^, ^41^). First, the researcher needs to specify (1) the models $H_{\cdot }$ under consideration, that is, the probability of data under the different parameter values that $H_{\cdot }$ allows, $p\left(\text{data}|\theta ,H_{\cdot }\right)$ (i.e., the likelihood), and (2) prior distributions for the model parameters *θ*, that is, the relative plausibility of the parameter values before observing the data, $p\left(\theta |H_{\cdot }\right)$. In the case of meta‐analyses, the data are usually represented by the observed effect sizes (*y*_*k*_) and their standard errors (*se*_*k*_) from k=1,…,K individual studies. For example, a fixed‐effect meta‐analytic model $H_{0}$ assuming absence of the mean effect (i.e., *μ* = 0) and no across‐study heterogeneity (i.e., *τ* = 0), can be defined as:

(1) $$\begin{array}{c} H_{0}:\mu =0,\;\tau =0\\ p\left(\text{data}|\theta_{0},H_{0}\right):y_{k}\sim \text{Normal}\left(0,{\mathrm{se}}_{k}\right), \end{array}$$

where *θ*_0_ denotes vector of parameters (*μ* and *τ*) belonging to the model $H_{0}$.

In contrast, a fixed‐effect meta‐analytic model $H_{1}$ assuming the presence of the mean effect (i.e., *μ* ≠ 0) needs to also specify a prior distribution for *μ*, *f*(.):

(2) $$\begin{array}{l} H_{1}:\mu \sim f\left(.\right),\;\tau =0\\ p\left(\text{data}|\theta_{1},H_{1}\right):y_{k}\sim \text{Normal}\left(\mu ,{\mathrm{se}}_{k}\right). \end{array}$$

Once the models have been specified, Bayes' rule dictates how the observed data update the prior distributions to posterior distributions, for each model separately:

(3) $$\begin{array}{l} p\left(\theta_{0}|H_{0},\text{data}\right)=\frac{p\left(\theta_{0}|H_{0}\right)\;p\left(\text{data}|\theta_{0},H_{0}\right)\;}{p\left(\text{data}|H_{0}\right)},\\ p\left(\theta_{1}|H_{1},\text{data}\right)=\frac{p\left(\theta_{1}|H_{1}\right)\;p\left(\text{data}|\theta_{1},H_{1}\right)}{p\left(\text{data}|H_{1}\right)}, \end{array}$$

where the denominators denote the marginal likelihood, that is, the average probability of the data under a particular model. Specifically, marginal likelihoods are obtained by integrating the likelihood over the prior distribution for the model parameters:

(4) $$\begin{array}{l} p(\text{data}|H_{0})=\int p\left(\text{data}|\theta_{0},H_{0}\right)\;p\left(\theta_{0}|H_{0}\right)\;d\theta_{0},\\ \;p\left(\text{data}|H_{1}\right)=\int p\left(\text{data}|\theta_{1},H_{1}\right)\;p\left(\theta_{1}|H_{1}\right)\;d\theta_{1}. \end{array}$$

Together with the likelihood, the prior parameter distribution determines the model's predictions. The marginal likelihood therefore quantifies a model's predictive performance in light of the observed data. Consequently, the marginal likelihood plays a pivotal role in model comparison and hypothesis testing.^42^ The ratio of two marginal likelihoods is known as the Bayes factor (BF),^43^, ^44^, ^45^, ^46^ and it indicates the extent to which one model outpredicts another; in other words, it grades the relative support that the models receive from the data. For example, the Bayes factor that assesses the relative predictive performance of the fixed‐effect meta‐analytic model $H_{0}:\mu =0$ to that of the fixed‐effect model $H_{1}:\mu \neq 0$ is

(5) $${\mathrm{BF}}_{10}=\frac{p\left(\text{data}|H_{1}\right)}{p\left(\text{data}|H_{0}\right)}.$$

The resulting BF_10_ represents the outcome of a Bayesian hypothesis test for the presence versus absence of an effect for the fixed‐effect meta‐analytic models. Unlike the *p*‐value in Neyman‐Pearson hypothesis testing, the BF value can be interpreted as a continuous measure of evidence. A BF_10_ value larger than 1 indicates support for the alternative hypothesis (in the nominator) and a value lower than 1 indicates support for the null hypothesis (in the denominator). As a general rule of thumb, Bayes factors between 1 and 3 (between 1 and 1/3) are regarded as anecdotal evidence, Bayes factors between 3 and 10 (between 1/3 and 1/10) are regarded as moderate evidence, and Bayes factors larger than 10 (smaller than 1/10) are regarded as strong evidence in favor of (against) a hypothesis (e.g., appendix I of Jeffreys^47^ and Lee & Wagenmakers^48^ p. 105). While this rule of thumb can aid interpretation, Bayes factors are inherently continuous measures of the strength of evidence and any attempt at discretization inevitably involves a loss of information.

Next, we incorporate the prior model probabilities that later allow us to weight the posterior model estimates by posterior probability of the considered models. It is common practice to divide the prior model probability equally across the different model types, that is, $p\left(H_{0}\right)=p\left(H_{1}\right)=1/2$.^36^, ^40^, ^49^, ^50^ To obtain the posterior model probabilities, we apply Bayes' rule one more time, now on the level of models instead of parameters:

(6) $$\begin{array}{l} p(H_{0}|\text{data})=\frac{p\left(H_{0}\right)\;p\left(\text{data}|H_{0}\right)}{p\left(\text{data}\right)},\\ \;p(H_{1}|\text{data})=\frac{p\left(H_{1}\right)\;p\left(\text{data}|H_{1}\right)}{p\left(\text{data}\right)}. \end{array}$$

The common denominator,

(7) $$p\left(\text{data}\right)=p\left(\text{data}|H_{0}\right)\;p\left(H_{0}\right)+p\left(\text{data}|H_{1}\right)\;p\left(H_{1}\right),$$

ensures that the posterior model probabilities sum to one.

The relative predictive performance of the rival models determines the update from prior to posterior model probabilities; in other words, models that predict the data well receive a boost in posterior probability, and models that predict the data poorly suffer a decline.^45^, ^51^ Thus, the Bayes factor quantifies the degree to which the data change the prior model odds to posterior model odds:

(8) $$\underset{\text{Bayes factor}}{\underbrace{\frac{p\left(\text{data}|H_{1}\right)}{p\left(\text{data}|H_{0}\right)}}}=\underset{\text{Posterior odds}}{\underbrace{\frac{p\left(H_{1}|\text{data}\right)}{p\left(H_{0}|\text{data}\right)}}}/\underset{\text{Prior odds}}{\underbrace{\frac{p\left(H_{1}\right)}{p\left(H_{0}\right)}}}.$$

We can combine the posterior parameter distributions from the two fixed‐effect meta‐analytic models by weighting the distributions according to the posterior model probabilities (e.g., Wrinch & Jeffreys,^46^ p. 387 and Jeffreys,^52^ p. 222). The resulting model‐averaged posterior distribution can be defined as a mixture distribution,

(9) $$p\left(\theta |\text{data}\right)=p\left(\theta_{0}|H_{0},\text{data}\right)\;p\left(H_{0}|\text{data}\right)+p\left(\theta_{1}|H_{1},\text{data}\right)\;p\left(H_{1}|\text{data}\right).$$

In RoBMA, the overall model ensemble is constructed from eight model types that represent the combination of the presence/absence of the effect, heterogeneity, and publication bias (modeled with two types of selection models in the original version of RoBMA^13^). With more than two models in play, Equations (5) and (9) can be expanded to accommodate the additional models. Specifically, the *inclusion Bayes factor* can be defined as a comparison between sets of models. For example, BF_10_ quantifies the evidence for presence versus absence of the effect by the change from prior to posterior odds for the set of models that include the effect versus the set of models that exclude the effect:

(10) $$\underset{\begin{array}{l} \text{Inclusion Bayes factor}\\ \;\text{for effect} \end{array}}{\underbrace{{\mathrm{BF}}_{10}}}=\underset{\begin{array}{l} \text{Posterior inclusion odds}\\ \text{for models assuming effect} \end{array}}{\underbrace{\frac{\sum_{i\in I}p\left(H_{i}|\text{data}\right)}{\sum_{j\in J}p\left(H_{j}|\text{data}\right)}}}/\underset{\begin{array}{l} \text{Prior inclusion odds}\\ \text{for models assuming effect} \end{array}}{\underbrace{\frac{\sum_{i\in I}p\left(H_{i}\right)}{\sum_{j\in J}p\left(H_{j}\right)}}},$$

where *i* ∈ *I* refers to models that include the effect and *j* ∈ *J* refers to models that exclude the effect.^38^, ^40^, ^†^ In the same way, we can also assess the relative predictive performance of any model compared to the rest of the ensemble.

Finally, the model‐averaged posterior distribution of *θ* is defined as a mixture distribution of the posterior distributions of *θ* from each model $H_{n}$ weighted by the posterior model probabilities,

(11) $$p\left(\theta |\text{data}\right)=\sum_{n=1}^{N}p\left(\theta_{n}|H_{n},\text{data}\right)\;p\left(H_{n}|\text{data}\right).$$

To complete the model‐averaged ensemble with multiple models corresponding to each component (e.g., two weight functions as a way of adjusting for publication bias in the original RoBMA), we maintain our prior indifference towards each of the hypotheses (e.g., presence/absence of the effect) by setting the prior model probabilities of all models that compose one of these two components to sum to 1/2. Often, the data contain enough information to assign posterior model probabilities to a class of similar models, largely washing out the effect of prior model probabilities on the model‐averaged posterior distribution. If the data do not contain enough information, the model‐averaged posterior distribution will be more affected by the choice of prior model probabilities. If researchers have diverging views on plausibility of different models, they can modify these prior model probabilities (e.g., by decreasing the prior model probabilities of fixed‐effect models, but see^50^).

In contrast to classical meta‐analytic statistics, the advantages of the Bayesian approach outlined above are that RoBMA can: (1) provide evidence for the absence of an effect (and therefore distinguish between “absence of evidence” and “evidence of absence”)^53^, ^54^; (2) update meta‐analytic knowledge sequentially, thus addressing recent concern about accumulation bias^55^; (3) incorporate expert knowledge; (4) retain and incorporate all uncertainty about parameters and models, without the need to make all‐or‐none choices; (5) emphasize the model outcomes that are most supported by the data, allowing it to flexibly adapt to scenarios with high heterogeneity and small sample sizes.

## 3 PUBLICATION BIAS ADJUSTMENT METHOD 1: SELECTION MODELS

One class of publication bias correction methods are selection models.^11^, ^12^, ^32^, ^56^, ^57^, ^‡^ In general, selection models estimate the relative probability that studies with *p*‐values within pre‐specified intervals were published as well as the corrected meta‐analytic effect size. In other words, they are directly accounting for the missing data, based on the modeled relation between statistical significance and probability of publication. Selection models differ mostly in the specified weight function (such as 3PSM and 4PSM^11^ and AK1 and AK2^20^), or are fit only to the statistically significant results (e.g., *p*‐curve^18^ and *p*‐uniform^19^).

Selection models based on *p*‐values are attractive for several reasons. First, the models provide a plausible account of the data generating process—statistically non‐significant studies are less likely to be published than statistically significant studies.^2^, ^4^, ^6^ Second, in recent simulation studies the unrestricted versions of selection models performed relatively well.^25^, ^27^

Selection models can be specified flexibly according to the assumed publication process. For example, we can distinguish between two‐sided selection (i.e., significant studies are published regardless of the direction of the effect) and one‐sided selection (only significant studies in the “correct” direction are preferentially reported). In the previous implementation of RoBMA, the selection models assumed two‐sided selection, either at a *p*‐value cutoff of 0.05 or also at a marginally significant cutoff of 0.10.^13^ In this paper, we extend RoBMA by adding 4 weight functions that encompass more ways in which the selection process might operate. The added weight functions assume one‐sided selection for positive effect sizes with cutoffs on significant, marginally significant, and/or *p*‐values corresponding to the expected effect size direction. Overall the six included weight functions are:

1. Two‐sided (already included in RoBMA)
  - *p*‐value cutoffs = 0.05;
  - *p*‐value cutoffs = 0.05 & 0.10.
2. One‐sided (new in RoBMA‐PSMA)
  - *p*‐value cutoffs = 0.05;
  - *p*‐value cutoffs = 0.025 & 0.05;
  - *p*‐value cutoffs = 0.05 & 0.50;
  - *p*‐value cutoffs = 0.025 & 0.05 & 0.50.

### 3.1 Example—Feeling the future

We illustrate this extended version of RoBMA on studies from the infamous 2011 article “Feeling the future: Experimental evidence for anomalous retroactive influences on cognition and affect.”^35^ Across a series of nine experiments, Bem^35^ attempted to show that participants are capable of predicting the future through the anomalous process of precognition. In response to a methodological critique, Bem and colleagues^60^ later conducted a meta‐analysis on the nine reported experiments in order to demonstrate that the experiments jointly contained strong support for existence of the effect. Publication of such an implausible result in the flagship journal of social psychology ignited an intense debate about replicability, publication bias, and questionable research practices in psychology.^7^

We analyze the data as described by Bem and colleagues^60^ in Table 1 with the updated version of RoBMA R package.^61^ For illustration, we specify the publication bias adjustment part with the six weight functions outlined above. We use the default prior distributions for the effect size and heterogeneity (standard normal and inverse‐gamma, respectively, as in the original version of RoBMA, see Appendix B (Data S1) for details). Internally, the package transforms the priors, the supplied Cohen's *d*, and their standard errors to the Fisher's *z* scale.^§^ The estimates are transformed back to Cohen's *d* scale for ease of interpretation. R code and data for reproducibility are available on OSF https://osf.io/fgqpc/.

**FIGURE 1:**
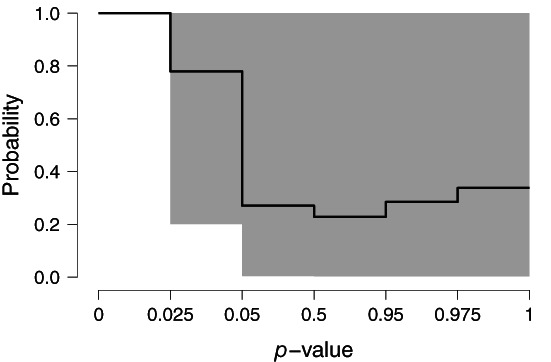
The model‐averaged weight function with 95% CI for Bem.^35^ Results are model‐averaged across the whole model ensemble, including models assuming no publication bias (*ω* = 1)

Our results do not provide notable evidence either for or against the presence of the anomalous effect of precognition: the model‐averaged Bayes factor equals BF_10_ = 1.91 and the posterior model‐averaged mean estimate of *μ* = 0.097, 95*%* CI [0.000, 0.232].^**^ Figure 1 shows posterior model‐averaged estimated weights with a re‐scaled *x*‐axis for easier readability. Because the meta‐analysis is based on only nine estimates, the uncertainty in the estimated weights is relatively high.

These results are an improvement from the original RoBMA implementation (with only 2 two‐sided weight functions) that showed strong support for the effect: BF_10_ = 97.89, *μ* = 0.149, 95*%* CI [0.053, 0.240]. The substantial difference in conclusions between the original RoBMA and the RoBMA with four additional weight functions is due to the inclusion of one‐sided selection models that seems to provide a better description for the Bem studies. More importantly, with the additional four weight functions RoBMA provides only moderate evidence for the effect even when adopting the *N*(0, 0.304^2^) prior distribution for effect size recommended by Bem and colleagues^60^: BF_10_ = 5.59, *μ* = 0.122, 95*%* CI [0.000, 0.234].

### 3.2 Limitations of selection models

While selection models have been shown to perform well in comparison to other methods in simulation studies,^25^, ^32^ they often insufficiently adjust for publication bias when applied to actual meta‐analytic data.^34^, ^64^ This discrepancy arises because the simulation studies assume that the selection model is an accurate reflection of the true data‐generating process; that is, the synthetic data obey a selection process that stipulates publication probability is (a) based solely on *p*‐values rather than on effect sizes; (b) based solely on a discretized *p*‐value interval, within which the probability of publication is constant. The simulation studies largely ignore the possibility of model misspecification and therefore provide an upper bound on model performance.^25^ A key strength of the Bayesian model‐averaging approach is that it can incorporate any number of models, increasing robustness and decreasing the potentially distorting effects of model misspecification. Therefore, we extend RoBMA with another method that adjusts for publication bias in an entirely different way—PET‐PEESE.^16^

## 4 PUBLICATION BIAS ADJUSTMENT METHOD 2: PET‐PEESE

A prominent class of alternative approaches to the selection models outlined above are methods that adjust for publication bias by adjusting for small‐study effects by estimating the relationship between effect sizes and their standard errors.^14^ The most well‐known approaches include trim and fill^17^ and PET‐PEESE.^16^ Here, we focus only on PET‐PEESE since its regression‐based framework, which fits the model to all observed studies, allows us to compare the model fit directly to the selection model‐based approaches.

PET‐PEESE method is an attractive addition to the RoBMA methodology since it often performs better than selection models in meta‐analytic applications^34^ (for applications in the field of ego depletion and antidepressant effectiveness see Carter and colleagues^65^ and Moreno and colleagues,^66^ respectively). PET‐PEESE is a conditional (two‐step) estimator composed of two models, PET model (i.e., Precision Effect Test) that is correctly specified when the effect is absent and PEESE model (i.e., Precision Effect Estimate with Standard Error) that provides a better approximation when the effect is present.^16^ The individual PET and PEESE models are the linear and the quadratic meta‐regression approximations, respectively, to the incidentally truncated selection model^16^ The choice between the PET and the PEESE model proceeds as follows: the test for the effect size coefficient based on PET (with *α* = 0.10 for model selection only) is used to decide whether the PET (*p* > *α*) or the PEESE (*p* < *α*) effect size estimator is employed.^67^

In order to add PET and PEESE models as a way of adjusting for publication bias with RoBMA, we modify them in the following way. Instead of following PET‐PEESE conditional selection of either PET or PEESE as proposed by Stanley & Doucouliagos,^16^ we include both PET and PEESE models, separately, in the RoBMA ensemble (alongside the weight functions model) and model‐average over the entire ensemble. Furthermore, instead of using an unrestricted weighted least squares estimator,^16^ we specify both fixed‐effects and random‐effects versions of these models, for consistency with the remaining RoBMA models. Consequently, the PET and PEESE models implemented in RoBMA correspond to meta‐regressions of effect size on either the standard errors or the variances with conventional fixed‐effects and random‐effect flavors (see Equation (2) in Appendix A (Data S1)).

In sum, we created a new RoBMA ensemble adjusting for publication bias using PET and PEESE models. Instead of using the model estimates conditionally, we model‐average across the fixed‐ and random‐effects PET and PEESE models assuming either absence or presence of the effect and the corresponding fixed‐ and random‐effects models without publication bias adjustment.

### 4.1 Example—Feeling the future

We revisit the Bem^35^ example. For illustration, we now specify only the PET and PEESE models as the publication bias adjustment part of the RoBMA ensemble (we include the six weight functions specified above in the next subsection). Again, we use the RoBMA package with the same default priors for effect size and heterogeneity; we assign Cauchy(0, 1) and Cauchy (0, 5) priors restricted to the positive range to the regression coefficients on standard errors and variances, respectively (see Appendix B (Data S1) for details).

**FIGURE 2:**
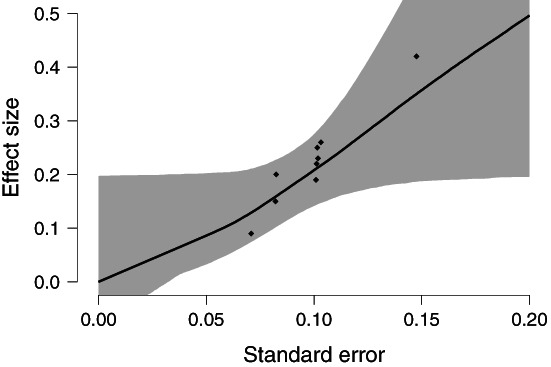
The relationship between the standard errors and model‐averaged effect size estimate with 95% CI for Bem.^35^ Results are model‐averaged across the entire model ensemble. Models assuming no publication bias have both PET and PEESE coefficients set to 0. Black diamonds correspond to the individual study estimates and standard errors

RoBMA version model‐averaging across the PET and PEESE models provides moderate evidence for the absence of an effect, BF_10_ = 0.226 (the reciprocal quantifying the evidence for the null hypothesis, BF_01_ = 4.42), with the posterior model‐averaged mean estimate *μ* = 0.013, 95*%* CI [−0.078, 0.197]. Figure 2 shows the estimated relationship between standard errors and effect sizes, where the effect size at standard error 0 corresponds to the posterior model‐averaged bias‐corrected estimate.

In this application, these results seem to provide an even better adjustment than the RoBMA version of selection models discussed previously. Furthermore, the RoBMA ensemble with PET‐PEESE models resolves a seeming inconsistency in the original conditional PET‐PEESE estimator. The frequentist PET model resulted in a significant negative effect size, *μ* = −0.182, *t*(7) = −3.65, *p* = 0.008, indicating that the effect size estimate from PEESE should be used, *μ* = 0.024, *t*(7) = 0.86, *p* = 0.418, which however is not notably different from zero. In addition, the RoBMA ensemble with PET‐PEESE does not provide evidence for precognition even under the more informed *N*(0, 0.304^2^) prior distribution for effect size recommended in Bem and colleagues^60^: BF_10_ = 0.670, *μ* = 0.028, 95*%* CI [−0.160, 0.215]. However, under this more informed prior, the data no longer provide moderate evidence against precognition.

### 4.2 Limitations of PET‐PEESE

While PET‐PEESE shows less bias and overestimation compared to other bias correction methods^34^ its key limitation is that the estimates can have very high variability. In simulation studies, PET‐PEESE can have high RMSE (root mean square error).^13^, ^25^, ^27^ Therefore, when PET‐PEESE based models are applied to an area of research for which they are ill‐suited, the resulting estimates may be inaccurate and unreliable. Stanley^67^ shows how the performance of PET‐PEESE can be especially problematic at very high levels of heterogeneity (*τ* ≥ 0.5), with low number of studies (i.e., *k* ≤ 10), and under uniformly low power.

## 5 COMBINING SELECTION MODELS AND PET‐PEESE

In order to obtain the best of both PET‐PEESE and selection models, we combine them into an overarching model: RoBMA‐PSMA. Specifically, RoBMA‐PSMA includes the 6 weight functions outlined in the section “Publication Bias Adjustment Methods 1: Selection Models” (assuming either presence or absence of the effect and heterogeneity, this yields 24 models) as well as the two PET and PEESE regression models outlined in the section “Publication Bias Adjustment Methods 2: PET‐PEESE” section (assuming either presence or absence of the effect and heterogeneity, this yields 8 models). We set the prior probability for the publication bias‐adjusted models to 0.5^13^ and divide this 0.5 probability equally across selection models and PET‐PEESE models (p. 47).^68^ Finally, adding models assuming absence of the publication bias (assuming either presence or absence of the effect and heterogeneity, this yields 4 models) results in a total of 24 + 8 + 4 = 36 models that together comprise RoBMA‐PSMA. The entire model ensemble is summarized in Table 1.

**TABLE 1:** RoBMA‐PSMA model ensemble together with prior parameter distributions (columns 1–3), prior model probabilities, (column 4), and posterior model probabilities (column 5) based on an application to the data from Bem^35^

*i*	Effect size	Heterogeneity	Publication bias	Prior prob.	Posterior prob.
1	*μ* = 0	*τ* = 0	None	0.125	0.000
2	*μ* = 0	*τ* = 0	*ω*_Two‐sided(0.05)_ ∼ CumDirichlet(1, 1)	0.010	0.000
3	*μ* = 0	*τ* = 0	*ω*_Two‐sided(0.1,0.05)_ ∼ CumDirichlet(1, 1, 1)	0.010	0.000
4	*μ* = 0	*τ* = 0	*ω*_One‐sided(0.05)_ ∼ CumDirichlet(1, 1)	0.010	0.012
5	*μ* = 0	*τ* = 0	*ω*_One‐sided(0.05,0.025)_ ∼ CumDirichlet(1, 1, 1)	0.010	0.034
6	*μ* = 0	*τ* = 0	*ω*_One‐sided(0.5,0.05)_ ∼ CumDirichlet(1, 1, 1)	0.010	0.001
7	*μ* = 0	*τ* = 0	*ω*_One‐sided(0.5,0.05,0.025)_ ∼ CumDirichlet(1, 1, 1, 1)	0.010	0.004
8	*μ* = 0	*τ* = 0	$\mathrm{PET}\sim \text{Cauchy}{\left(0,1\right)}_{\left[0,\infty \right]}$	0.031	0.281
9	*μ* = 0	*τ* = 0	$\text{PEESE}\sim \text{Cauchy}{\left(0,5\right)}_{\left[0,\infty \right]}$	0.031	0.254
10	*μ* = 0	*τ* ∼ InvGamma(1, 0.15)	None	0.125	0.000
11	*μ* = 0	*τ* ∼ InvGamma(1, 0.15)	*ω*_Two‐sided(0.05)_ ∼ CumDirichlet(1, 1)	0.010	0.000
12	*μ* = 0	*τ* ∼ InvGamma(1, 0.15)	*ω*_Two‐sided(0.1,0.05)_ ∼ CumDirichlet(1, 1, 1)	0.010	0.000
13	*μ* = 0	*τ* ∼ InvGamma(1, 0.15)	*ω*_One‐sided(0.05)_ ∼ CumDirichlet(1, 1)	0.010	0.014
14	*μ* = 0	*τ* ∼ InvGamma(1, 0.15)	*ω*_One‐sided(0.05,0.025)_ ∼ CumDirichlet(1, 1, 1)	0.010	0.020
15	*μ* = 0	*τ* ∼ InvGamma(1, 0.15)	*ω*_One‐sided(0.5,0.05)_ ∼ CumDirichlet(1, 1, 1)	0.010	0.006
16	*μ* = 0	*τ* ∼ InvGamma(1, 0.15)	*ω*_One‐sided(0.5,0.05,0.025)_ ∼ CumDirichlet(1, 1, 1, 1)	0.010	0.010
17	*μ* = 0	*τ* ∼ InvGamma(1, 0.15)	$\mathrm{PET}\sim \text{Cauchy}{\left(0,1\right)}_{\left[0,\infty \right]}$	0.031	0.021
18	*μ* = 0	*τ* ∼ InvGamma(1, 0.15)	$\text{PEESE}\sim \text{Cauchy}{\left(0,5\right)}_{\left[0,\infty \right]}$	0.031	0.017
19	*μ* ∼ Normal(0, 1)	*τ* = 0	None	0.125	0.051
20	*μ* ∼ Normal(0, 1)	*τ* = 0	*ω*_Two‐sided(0.05)_ ∼ CumDirichlet(1, 1)	0.010	0.007
21	*μ* ∼ Normal(0, 1)	*τ* = 0	*ω*_Two‐sided(0.1,0.05)_ ∼ CumDirichlet(1, 1, 1)	0.010	0.031
22	*μ* ∼ Normal(0, 1)	*τ* = 0	*ω*_One‐sided(0.05)_ ∼ CumDirichlet(1, 1)	0.010	0.030
23	*μ* ∼ Normal(0, 1)	*τ* = 0	*ω*_One‐sided(0.05,0.025)_ ∼ CumDirichlet(1, 1, 1)	0.010	0.035
24	*μ* ∼ Normal(0, 1)	*τ* = 0	*ω*_One‐sided(0.5,0.05)_ ∼ CumDirichlet(1, 1, 1)	0.010	0.018
25	*μ* ∼ Normal(0, 1)	*τ* = 0	*ω*_One‐sided(0.5,0.05,0.025)_ ∼ CumDirichlet(1, 1, 1, 1)	0.010	0.022
26	*μ* ∼ Normal(0, 1)	*τ* = 0	$\mathrm{PET}\sim \text{Cauchy}{\left(0,1\right)}_{\left[0,\infty \right]}$	0.031	0.047
27	*μ* ∼ Normal(0, 1)	*τ* = 0	$\text{PEESE}\sim \text{Cauchy}{\left(0,5\right)}_{\left[0,\infty \right]}$	0.031	0.046
28	*μ* ∼ Normal(0, 1)	*τ* ∼ InvGamma(1, 0.15)	None	0.125	0.007
29	*μ* ∼ Normal(0, 1)	*τ* ∼ InvGamma(1, 0.15)	*ω*_Two‐sided(0.05)_ ∼ CumDirichlet(1, 1)	0.010	0.001
30	*μ* ∼ Normal(0, 1)	*τ* ∼ InvGamma(1, 0.15)	*ω*_Two‐sided(0.1,0.05)_ ∼ CumDirichlet(1, 1, 1)	0.010	0.003
31	*μ* ∼ Normal(0, 1)	*τ* ∼ InvGamma(1, 0.15)	*ω*_One‐sided(0.05)_ ∼ CumDirichlet(1, 1)	0.010	0.005
32	*μ* ∼ Normal(0, 1)	*τ* ∼ InvGamma(1, 0.15)	*ω*_One‐sided(0.05,0.025)_ ∼ CumDirichlet(1, 1, 1)	0.010	0.005
33	*μ* ∼ Normal(0, 1)	*τ* ∼ InvGamma(1, 0.15)	*ω*_One‐sided(0.5,0.05)_ ∼ CumDirichlet(1, 1, 1)	0.010	0.003
34	*μ* ∼ Normal(0, 1)	*τ* ∼ InvGamma(1, 0.15)	*ω*_One‐sided(0.5,0.05,0.025)_ ∼ CumDirichlet(1, 1, 1, 1)	0.010	0.004
35	*μ* ∼ Normal(0, 1)	*τ* ∼ InvGamma(1, 0.15)	$\mathrm{PET}\sim \text{Cauchy}{\left(0,1\right)}_{\left[0,\infty \right]}$	0.031	0.004
36	*μ* ∼ Normal(0, 1)	*τ* ∼ InvGamma(1, 0.15)	$\text{PEESE}\sim \text{Cauchy}{\left(0,5\right)}_{\left[0,\infty \right]}$	0.031	0.004

*Note*: *μ* corresponds to the effect size parameter, *τ* to the heterogeneity parameter, *ω* to the weight parameters with an appropriate selection process (either one or two‐sided with given cutoffs), PET to the regression coefficient on the standard errors, and PEESE to the regression coefficient on variances. All prior distributions are specified on the Cohen's *d* scale.

As mentioned above, RoBMA‐PSMA draws inference about the data by considering all models simultaneously. Specific inferences can be obtained by interrogating the model ensemble and focusing on different model classes. Concretely, the evidence for presence versus absence of the effect is quantified by the inclusion Bayes factor BF_10_ (Equation (10)) obtained by comparing the predictive performance of models assuming the effect is present (i=19,…,36 in Table 1) to that of models assuming the effect is absent (j=1,…,18 in Table 1). In the Bem example, substituting the prior and posterior model probabilities from Table 1 yields BF_10_ = 0.479. This Bayes factor indicates that the posterior inclusion odds for the models assuming the effect is present are slightly lower than the prior inclusion odds. In other words, models assuming that the effect is absent predicted the data about 1/0.479 ≈ 2.09 times better than models assuming the effect is present. This result aligns with the common scientific understanding of nature, which the presence of precognition would effectively overturn.

The remaining Bayes factors are calculated similarly. The Bayes factor for the presence versus absence of heterogeneity, BF_*rf*_, compares the predictive accuracy of models assuming heterogeneity (i=10,…,18and28,…36 in Table 1) with models assuming homogeneity (j=1,…,9and19,…27 in Table 1). With BF_*rf*_ = 0.144, the data disfavor the models assuming heterogeneity; that is, the data are BF_*fr*_ = 1/0.144 ≈ 6.94 times more likely to occur under homogeneity than under heterogeneity. Analogously, the Bayes factor for the presence versus absence of publication bias, BF_*pb*_, compares predictive performance of models assuming publication bias is present (i=2,…,9,11,…18,20…27,and29,…36 in Table 1) to that of models assuming publication bias is absent (*j* ∈ 1, 10, 19, and 28 in Table 1). Here, the results show strong support in favor of the models assuming publication bias is present, BF_*pb*_ = 16.31.

Model‐averaging can also be used to compare the different types of publication bias adjustment methods. Specifically, the predictive performance of the selection models (*i* = 2, …, 7, 11, …, 16, 20, …, 25, and 29, …, 34) may be contrasted to that of the PET‐PEESE models (*j* = 8, 9, 17, 18, 26, 27, 35, and 36), yielding BF = 0.397 (cf. Equation (10)); this result indicates that the posterior probability increases more for the PET‐PEESE models (0.25 → 0.675) than it does for the selection models (0.25 → 0.268), especially selection model assuming one‐sided selection that were better supported by the data (0.166 → 0.225) than the two‐sided selection models (0.083 → 0.043). However, this Bayes factor only modestly favors the PET‐PEESE models, and consequently the results from the selection models also contribute substantially towards the final posterior model‐averaged estimate.

Predictive performance of individual models may be contrasted to that of the rest of the ensemble (cf. Equation (10)). For Bem,^35^ the data most strongly supported the PET and PEESE models assuming no effect and no heterogeneity, BF = 12.13 and BF = 10.57, respectively—the corresponding model probabilities increased from 0.031 to 0.280 and 0.255.

The posterior model‐averaged effect size estimate *μ* is obtained by combining the 36 estimates across all models in the ensemble, weighted according to their posterior model probabilities. Some of the models assume the effect is absent, and concentrate all prior probability mass on *μ* = 0; therefore, the model‐averaged posterior distribution is a combination of a “spike” at 0 and a mixture of continuous probability densities that correspond to the alternative models. When the alternative models are strongly supported by the data, the impact of the spike is minimal and the model‐averaged posterior distribution reduces to a mixture of continuous densities. In the Bem^35^ example, RoBMA‐PSMA gives a posterior model‐averaged mean estimate *μ* = 0.038, 95*%* CI [−0.034, 0.214] (cf. Equation (9)). The posterior model‐averaged estimates for the remaining parameters, for example, the heterogeneity estimate *τ* or the publication weights *ω*, are obtained similarly.

The overall results would, again, remain similar even when using the Bem and colleagues'^60^ more informed prior distribution for effect size, *N*(0, 0.304^2^): BF_10_ = 1.41, *μ* = 0.067, 95*%* CI [−0.111, 0.226]. These results are in line with failed replication studies,^69^, ^70^, ^71^, ^72^ evidence of QRPs,^73^, ^74^, ^75^ and common sense^76^; see also.^60^, ^77^, ^78^, ^79^, ^80^

## 6 EVALUATING ROBMA THROUGH REGISTERED REPLICATION REPORTS

Kvarven and colleagues^34^ compared the effect size estimates from 15 meta‐analyses of psychological experiments to the corresponding effect size estimates from Registered Replication Reports (RRR) of the same experiment.^††^ RRRs are accepted for publication independently of the results and should be unaffected by publication bias. The original meta‐analyses reveal considerable heterogeneity; thus, any single RRR is unlike to directly correspond to the true mean meta‐analytic effect size. As a result, the comparison of meta‐analysis results to RRRs will inflate RMSE and can be considered a highly conservative way of evaluating bias detection methods. However, when averaged over 15 RRRs, we would expect little systematic net heterogeneity and a notable reduction in aggregate bias. In this way, average bias adjusted estimates should randomly cluster around the average of the RRR estimates. In other words, we would expect little overall bias, relative to RRRs. Hence, the comparison to RRRs can be used to gauge the performance of publication bias adjustment methods, while keeping in mind that the studies are heterogeneous and limited in number. Kvarven and colleagues^34^ found that conventional meta‐analysis methods resulted in substantial overestimation of effect size. In addition, Kvarven and colleagues^34^ examined three popular bias detection methods: trim and fill (TF),^17^ PET‐PEESE,^16^ and 3PSM.^11^, ^56^ The best performing method was PET‐PEESE; however, its estimates still have notable RMSE.

Here we use the data analyzed by Kvarven and colleagues^34^ as one way of comparing the performance of RoBMA‐PSMA in relation to a series of alternative publication bias correction methods. These methods include those examined by Kvarven and colleagues^34^—PET‐PEESE, 3PSM, and TF—as well as a set of seven other methods^27^: 4PSM,^11^ AK1 and AK2,^20^ *p*‐curve,^18^ *p*‐uniform^19^ WAAP‐WLS,^22^ and endogenous kink (EK).^21^ For completeness, we also show results for the original implementation of RoBMA‐old.^13^ The RoBMA‐old, 3PSM, 4PSM, AK1, AK2, *p*‐curve, and *p*‐uniform can be viewed as selection models operating on *p*‐values that mostly differ in thresholds of the weight function and estimation algorithm. The PET‐PEESE, TF, and EK can be viewed as methods correcting for publication bias based on relationship between effect sizes and standard errors. Finally, RoBMA‐PSMA is a method that combines both types of publication bias corrections.

Following Kvarven and colleagues,^34^ we report all meta‐analytic estimates on the Cohen's *d* scale, with one exception for a meta‐analysis that used Cohen's *q* scale. As in the Bem example, RoBMA internally transforms effect sizes from the Cohen's *d* scale to the Fisher *z* scale.^‡‡^ Each method is evaluated on the following five metrics (cf.^34^): (1) false positive rate (FPR), that is, the proportion of cases where the RRR fails to reject the null hypothesis (i.e., *p* > 0.05) whereas the meta‐analytic method concludes that the data offer support for the presence of the effect (i.e., *p* < 0.05 or BF_10_ > 10); (2) false negative rate (FNR), that is, the proportion of cases where the RRR rejects the null hypothesis (i.e., *p* < 0.05) whereas the meta‐analytic method fails to reject the null/finds evidence for the absence of the effect (i.e., *p* > 0.05 or BF_10_ < 1/10)^§§^; (3) overestimation factor (OF), that is, the meta‐analytic mean effect size divided by the RRR mean effect size; (4) bias, that is, the mean difference between the meta‐analytic and RRR effect size estimates; and (5) root mean square error (RMSE), that is, the square root of the mean of squared differences between the meta‐analytic and RRR effect size estimates. Note that when evaluating the methods' qualitative decisions (i.e., FPR and FNR), the RoBMA methods do not necessarily lead to a strong claim about the presence or absence of the effect; in the Bayesian framework, there is no need to make an all‐or‐none decision based on weak evidence, and here we have defined an in‐between category of evidence that does not allow a confident conclusion (i.e., Undecided, 1/10 < BF_10_ < 10; for a discussion on the importance of this in‐between category see Robinson^53^). Furthermore, selecting a different significance level or Bayes factor thresholds would lead to different false positive and false negative rates.

The main results are summarized in Table 2. Evaluated across all metrics simultaneously, RoBMA‐PSMA generally outperforms the other methods. RoBMA‐PSMA has the lowest bias, the second‐lowest RMSE, and the second lowest overestimation factor. The only methods that perform better in one of the categories (i.e., AK2 with the lowest overestimation factor; PET‐PEESE and EK with the second and third lowest bias, respectively), showed considerably larger RMSE, and AK2 converged in only 5 out of 15 cases. Furthermore, RoBMA‐PSMA resulted in conclusions that are qualitatively similar to those from the RRR studies. Specifically, for cases where the RRR was statistically significant, RoBMA‐PSMA never showed evidence for the absence of the effect (i.e., FNR = 0/8 = 25%) but often did not find compelling evidence for the presence of the effect either (i.e., Undecided = 6/8 = 75%). Furthermore, for cases where the RRR was not statistically significant, RoBMA‐PSMA showed evidence for the presence of the effect only once (i.e., FPR = 1/7 ≈ 14.3%) and did not find compelling evidence for the absence of the effect in the remaining meta‐analyses (i.e., Undecided = 6/7 ≈ 85.7%). After adjusting for publication selection bias with RoBMA, the original meta‐analyses often did not contain sufficient evidence for firm conclusions about the presence versus absence of the effect.^***^ This highlights the oft‐hidden reality that the data at hand do not necessarily warrant strong conclusions about the phenomena under study; consequently, a final judgment needs to be postponed until more data accumulates.

**TABLE 2:** Performance of 13 publication bias correction methods for the Kvarven and colleagues^34^ test set comprised of 15 meta‐analyses and 15 corresponding “Gold Standard” registered replication reports (RRR)

Method	FPR/Undecided	FNR/Undecided	OF	Bias	RMSE
RoBMA‐PSMA	0.143/0.857	0.000/0.750	1.160	0.026	0.164
*AK2*	*0.000/—*	*0.250/—*	*1.043*	*−0.070*	*0.268*
PET‐PEESE	0.143/—	0.500/—	1.307	0.050	0.256
EK	0.143/—	0.500/—	1.399	0.065	0.283
RoBMA‐old	0.714/0.286	0.000/0.000	2.049	0.171	0.218
4PSM	0.714/—	0.500/—	1.778	0.127	0.268
3PSM	0.714/—	0.125/—	2.193	0.195	0.245
*TF*	*0.833/—*	*0.000/—*	*2.315*	*0.206*	*0.259*
AK1	0.857/—	0.000/—	2.352	0.221	0.264
*p*‐uniform	0.500/—	0.429/—	2.375	0.225	0.288
*p*‐curve			2.367	0.223	0.289
WAAP‐WLS	0.857/—	0.125/—	2.463	0.239	0.295
Random Effects (DL)	1.000/—	0.000/—	2.586	0.259	0.310

*Note*: The results in *italic* are conditional on convergence: trim and fill did not converge in one case and AK2 did not converge in 10 cases. The rows are ordered based on combined log scores performance of the abs(log(OF)), abs(Bias), and RMSE (not shown).

Abbreviations: FNR/Undecided, false negative rate/undecided evidence under an effect; FPR/Undecided, false positive rate/undecided evidence under no effect; OF, overestimation factor; RMSE, root mean square error.

**FIGURE 3:**
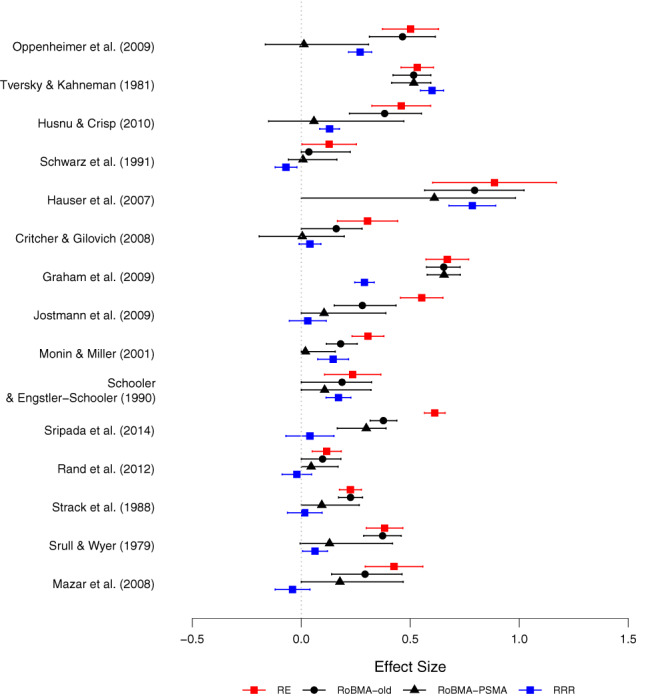
Effect size estimates with 95% CIs from a random‐effects meta‐analysis, three RoBMA models, and the RRR for the 15 experiments included in Kvarven et al.^34^ Estimates are reported on the Cohen's *d* scale [Colour figure can be viewed at wileyonlinelibrary.com]

Figure 3 shows the effect size estimates from the RRRs for each of the 15 cases, together with the estimates from a random effects meta‐analysis and the posterior model‐averaged estimates from RoBMA and RoBMA‐PSMA (figures comparing all methods for each RRR are available in the “Kvarven et al/estimates figures” folder in the online supplementary materials at https://osf.io/fgqpc/files/). Because RoBMA‐PSMA corrects for publication bias, its estimates are shrunken towards zero. In addition, the estimates from RoBMA‐PSMA also come with wider credible intervals (reflecting the additional uncertainty about the publication bias process) and are generally closer to the RRR results. The most anomalous case concerns the Graham and colleagues^85^ study, where all four methods yield similar intervals, but the RRR shows an effect size that is twice as small. This result may be due to cultural differences and the choice of the social or economic dimension that all contributed to heterogeneity in the original meta‐analysis.^86^

Appendix C (Data S1) demonstrates robustness of our findings by estimating RoBMA under different parameter prior distributions. Appendix D (Data S1) presents a non‐parametric bootstrap analysis of the RRR comparison, showing high uncertainty in the FPR and FNR, but qualitatively robust conclusions about the overestimation factor, bias, and RMSE. Appendix E (Data S1) demonstrates that our findings are not a result of a systematic underestimation of effect sizes by estimating RoBMA on 28 sets of Registered Replication Reports from Many Labs 2.^87^

## 7 EVALUATING ROBMA THROUGH SIMULATION STUDIES

We evaluate the performance of the newly developed RoBMA methods using simulation studies.^27^ As in Hong & Reed,^27^ we tested the methods in four simulation environments, namely those developed by Stanley and colleagues^22^ (SD&I), Alinaghi & Reed^88^ (A&R), Bom & Rachinger^21^ (B&R), and Carter and colleagues^25^ (CSG&H). These environments differ in terms of assumptions concerning effect sizes, heterogeneity, sample sizes, and publication bias; moreover, CSG&H include questionable research practices (QRP).^9^ Briefly, the SD&I environment relates to the settings usually found in medicine where a difference between two groups is assessed with either continuous or dichotomous outcomes. The A&R environment is similar to the settings encountered in economics and business and consists of relationships between two continuous variables with multiple estimates originating from a single study. The B&R environment considers situations where regression coefficients are routinely affected by an omitted‐variables bias. The CSG&H environment is most typical for psychology with effect sizes quantifying differences in a continuous measure between groups. For each condition from each of the four simulation environments, Hong & Reed^27^ generated 3000 synthetic data sets that were then analyzed by all of the competing methods.

Here we used the code, data, and results publicly shared by Hong & Reed.^27^ Because our Bayesian methods require computationally intensive Markov chain Monte Carlo estimation, we used only 300 synthetic data sets per condition (10% of the original replications).^†††^ Nevertheless, our simulations still required ~25 CPU years to complete. A detailed description of the simulation environments (consisting of a total of 1620 conditions) and the remaining methods can be found in Hong & Reed^27^ and the corresponding original simulation publications. We compared the performance of RoBMA‐PSMA to all methods used in the previous section. See Supplementary Materials at https://osf.io/bd9xp/ for comparison of methods after removing 5% of the most extreme estimates from each method, as done by Hong & Reed,^27^ with the main difference being an improved performance of AK1 and AK2.

Tables 3 and 4 summarize the aggregated results for mean square error (MSE) and bias, respectively, separately for each simulation environment. Although no single estimator dominates across all simulation environments and criteria, RoBMA‐PSMA is at or near the top in most cases. The exception is that RoBMA‐PSMA produces below‐average performance in the CSG&H environment. Tables S7 and S8 in Appendix F (Data S1) show that RoBMA‐PSMA overcorrects the effect size estimates and performs relatively poorly only in conditions with *p*‐hacking strong enough to introduce significant skew in the distributions of effect sizes.^‡‡‡^

**TABLE 3:** Ordered performance of the methods according to MSE for each simulation environment. Rank 1 has the lowest MSE. See text for details

Rank	SD&I	MSE	A&R	MSE	B&R	MSE	CSG&H	MSE
1	RoBMA‐PSMA	0.009	RoBMA‐PSMA	0.222	RoBMA‐PSMA	0.098	RoBMA‐old	0.012
2	*AK2* ^a^	*0.013*	TF	0.273	*p‐uniform*	*0.185*	WAAP‐WLS	0.018
3	RoBMA‐old	0.017	*AK2* ^a^	*0.277*	WAAP‐WLS	0.193	TF	0.022
4	TF	0.025	RoBMA‐old	0.327	RoBMA‐old	0.221	*3PSM*	*0.023*
5	WAAP‐WLS	0.025	4PSM	0.365	TF	0.321	PET‐PEESE	0.027
6	PET‐PEESE	0.028	AK1^a^	0.389	EK	0.375	*p*‐uniform	0.028
7	EK	0.031	Random Effects (DL)	0.511	PET‐PEESE	0.378	*4PSM*	*0.031*
8	Random Effects (DL)	0.034	3PSM	0.511	4PSM	0.492	EK	0.033
9	*p*‐uniform	0.050	WAAP‐WLS	0.546	3PSM	0.493	RoBMA‐PSMA	0.036
10	3PSM	0.238	PET‐PEESE	0.605	Random Effects (DL)	0.526	Random Effects (DL)	0.046
11	*p*‐curve	1.228	EK	0.760	*p*‐curve	0.850	*p*‐curve	0.075
12	4PSM	3.375	*p*‐curve	3.514	AK1^a^	2.806	AK1^a^	0.280
13	AK1^a^	6.231	*p*‐uniform	3.621	*AK2* ^a^	*5.816*	*AK2* ^a^	*2.849*

*Note*: Methods in *italic* converged in fewer than 90% repetitions in a given simulation environment.

^a^ The performance difference in terms of MSE for AK1 and AK2 between our implementation and that of Hong & Reed^27^ is due to the fact that we did not omit the 5% most extreme estimates.

**TABLE 4:** Ordered performance of the methods according to bias for each simulation environment. Rank 1 has the lowest bias. See text for details

Rank	SD&I	Bias	A&R	Bias	B&R	Bias	CSG&H	Bias
1	*AK2*	*0.029*	RoBMA‐PSMA	0.159	EK	0.095	PET‐PEESE	0.059
2	RoBMA‐PSMA	0.034	*AK2*	*0.207*	*AK2*	*0.105*	WAAP‐WLS	0.062
3	3PSM	0.040	EK	0.221	4PSM	0.108	RoBMA‐old	0.064
4	PET‐PEESE	0.049	PET‐PEESE	0.259	RoBMA‐PSMA	0.121	AK1	0.067
5	EK	0.053	WAAP‐WLS	0.266	PET‐PEESE	0.129	EK	0.072
6	RoBMA‐old	0.062	TF	0.288	3PSM	0.156	*3PSM*	*0.081*
7	AK1	0.082	4PSM	0.302	WAAP‐WLS	0.189	TF	0.091
8	WAAP‐WLS	0.083	RoBMA‐old	0.354	RoBMA‐old	0.228	*4PSM*	*0.096*
9	TF	0.088	AK1	0.397	TF	0.240	*p*‐uniform	0.106
10	4PSM	0.088	3PSM	0.475	AK1	0.277	RoBMA‐PSMA	0.110
11	Random effects (DL)	0.108	Random effects (DL)	0.556	Random effects (DL)	0.363	*AK2*	*0.117*
12	*p*‐uniform	0.147	*p*‐curve	1.530	*p*‐uniform	0.374	*p*‐curve	0.118
13	*p*‐curve	0.422	*p*‐uniform	1.555	*p*‐curve	0.522	Random effects (DL)	0.150

*Note*: Methods in *italic* converged in fewer than 90% repetitions in a given simulation environment.

Following Hong & Reed,^27^ Table 5 averages performance across all four simulation environments. While the results confirm that RoBMA‐PSMA performs the best with regard to type I error rates and coverage, it is important to note that both the coverage and error rate were far above the nominal levels. The results also appear favorable to AK2, as it has the lowest bias in SD&I environment and the second lowest biases in the A&R and B&R environments. However, AK2 failed to converge in over 10% of these simulated meta‐analyses. Even when AK2 converges, its MSE in the B&R and CSG&H environments is relatively large.

**TABLE 5:** Aggregated results over all simulation conditions from Hong & Reed^27^

Rank	Rank (Bias)	Bias	Rank (MSE)	MSE	Rank ($\left|\text{Coverage}-0.95\right|$)	$\left|\text{Coverage}-0.95\right|$	Rank (ERR)	ERR
1	EK	0.079	RoBMA‐PSMA	0.054	*AK2* ^a^	*0.167*	RoBMA‐PSMA	0.093
2	PET‐PEESE	0.083	RoBMA‐old	0.085	RoBMA‐PSMA	0.172	*AK2* ^a^	*0.129*
3	*AK2* ^a^	*0.099*	WAAP‐WLS	0.085	3PSM	0.213	EK	0.257
4	RoBMA‐PSMA	0.099	TF	0.121	4PSM	0.265	3PSM	0.259
5	4PSM	0.103	PET‐PEESE	0.149	PET‐PEESE	0.306	PET‐PEESE	0.286
6	3PSM	0.105	EK	0.155	EK	0.307	4PSM	0.290
7	WAAP‐WLS	0.110	*p*‐uniform	0.161	RoBMA‐old	0.317	RoBMA‐old	0.485
8	RoBMA‐old	0.121	Random Effects (DL)	0.203	WAAP‐WLS	0.319	WAAP‐WLS	0.525
9	TF	0.141	3PSM	0.223	AK1^a^	0.341	AK1^a^	0.573
10	AK1^a^	0.143	*p*‐curve	0.623	TF	0.407	*p*‐uniform	0.585
11	Random Effects (DL)	0.217	4PSM	0.851	Random Effects (DL)	0.510	TF	0.597
12	*p*‐uniform	0.230	AK1^a^	2.258	*p*‐uniform	0.576	Random Effects (DL)	0.649
13	*p*‐curve	0.336	*AK2*	*3.316*	*p*‐curve		*p*‐curve	

*Note*: Ranking and values of aggregated bias, mean square error (MSE), absolute difference from 0.95 CI coverage ($\left|\text{Coverage}-0.95\right|$), and type I error rate (ERR) averaged across all simulation environments in (^27^; the type I error rate for RoBMA methods is based on BF > 10). Methods in *italic* converged in fewer than 90% repetitions in a given simulation environment.

^a^ The performance difference in terms of MSE for AK1 and AK2 between our implementation and that of Hong & Reed^27^ is due to the fact that we did not omit the 5% most extreme estimates.

It should be noted that the averaging operation is valid only for coverage and type I error rates, as these are fully comparable across the different simulation environments. In contrast, bias and MSE cannot be directly averaged or aggregated, as these are based on very different effect‐size metrics^27^; for instance, the best method in A&R environment has five times the bias as the best method in SD&I environment. In order to make the metrics commensurate, we employ a relative order‐preserving logarithmic transformation to obtain an average ranking across these four different simulation environments^89^ (1 corresponds to the best relative performance, 0 to the worst relative performance).^§§§^ Table 6 displays the average relative ranks of bias and MSE for these alternative methods across all simulation environments. RoBMA‐PSMA is ranked highest according to MSE and type I error rates, and is the second best according to both bias and confidence interval coverage. Again, the closest competition appears to come from AK2, but AK2 often does not converge and may yield high MSEs—see Table 3. Table 5 also shows that RoBMA‐PSMA does well when bias and MSE are simply averaged across these simulations, but those comparisons need to be interpreted with caution.

**TABLE 6:** Ordered performance of the methods across simulation environments according to log scoring rule

Rank	Bias	Log score (Bias)	MSE	Log score (MSE)
1	*AK2*	*0.801*	RoBMA‐PSMA	0.831
2	RoBMA‐PSMA	0.801	RoBMA‐old	0.682
3	EK	0.778	TF	0.519
4	PET‐PEESE	0.746	WAAP‐WLS	0.504
5	WAAP‐WLS	0.616	*AK2*	*0.392*
6	3PSM	0.615	PET‐PEESE	0.369
7	4PSM	0.602	EK	0.327
8	RoBMA‐old	0.579	*p*‐uniform	0.324
9	AK1	0.515	3PSM	0.316
10	TF	0.500	4PSM	0.315
11	Random effects (DL)	0.329	Random effects (DL)	0.310
12	*p*‐uniform	0.304	AK1	0.183
13	*p*‐curve	0.242	*p*‐curve	0.114

*Note*: Methods in *italic* converged in less than 90% repetitions.

## 8 CONCLUDING COMMENTS

We have extended the robust Bayesian meta‐analytic framework with one‐sided weight functions and PET‐PEESE regression models. This extension allows researchers to draw inferences using a multitude of otherwise competing approaches (i.e., selection models based on *p*‐values and models estimating the relationship between effect sizes and standard errors). Consequently, researchers interested in obtaining the best possible adjusted meta‐analytic effect size estimate do not need to speculate about the type of publication bias in order to select the best method for their setting. Instead, RoBMA weights its inference in proportion to how well each method accounts for the data.

The extended version of RoBMA resolves the tension between the selection models and PET‐PEESE. Furthermore, we demonstrated that RoBMA‐PSMA outperforms previous methods when applied to actual meta‐analyses for which a gold standard is available.^34^ Finally, the new RoBMA methods performed well in simulation studies. However, it is important to note that RoBMA‐PSMA did not perform well in simulation settings of Carter and colleagues^25^ with prominent *p*‐hacking where it overcorrected the effect sizes.

The RoBMA framework can be further extended in multiple ways: to account for multilevel structures, to estimate within study clusters, to deal with multivariate outcomes, and to include of explanatory variables. Many of those extensions will, however, increase computational complexity, making them practically unfeasible for selection models. Therefore, further research is need in developing efficient algorithms or approximations that will allow the further extensions, currently unachievable under the RoBMA‐PSMA framework.

Out of the remaining methods, *p*‐curve, *p*‐uniform, and random effects meta‐analysis were dominated by the other estimators, and AK2 failed to converge in many cases. Overall, Bayesian model‐averaging greatly improved both PET‐PEESE and selection models: RoBMA‐PSMA reduces PET‐PEESE's MSE and bias as well as the selection models' MSE. Importantly, RoBMA‐PSMA takes uncertainty about the type of publication bias into account and combines the best of the two worlds. Even though RoBMA outperforms other methods in many cases in both the simulation study and the comparison of meta‐analyses and registered replication reports, it should be considered merely a new tool in the toolbox of publication selection bias detection.

In cases where the data generating process is known and depending on the metric that researchers want to optimize (e.g., bias vs. RMSE) an appropriate method can be selected via the results from our simulation study or the meta‐showdown explorer https://tellmi.psy.lmu.de/felix/metaExplorer/. If there is considerable uncertainty about the data generating process, we believe that RoBMA is a sensible default. Nevertheless, researchers may wish to check the conclusions of RoBMA against methods that are not part of the RoBMA ensemble, such as WAAP‐WLS. As there is no principled way of averaging these methods with RoBMA,^****^ researchers should view these comparisons as sensitivity analyses. If alternative methods come to the same conclusions as RoBMA, this suggests that the results are robust; If alternative methods come to a qualitatively different conclusion, this suggests that the results are fragile; in this case, we recommend a more in‐depth consideration of the data‐model relationship, and a transparent report that the conclusions vary based on the selected meta‐analytic technique.

We believe that the extended version of RoBMA with the outlined default prior distributions presents a reasonable setup for anyone interested in performing a meta‐analysis. However, the RoBMA framework is flexible and allows researchers to specify different prior distributions for any of the model parameters or include/exclude additional models (see “Appendix B: Specifying Different Priors” in,^90^ or many of the R package vignettes). Consequently, researchers with substantial prior knowledge can test more specific hypotheses than those specified with the default model ensemble^39^, ^50^, ^91^ or incorporate prior knowledge about the research environment. For instance, when prior research has established that the effect of interest shows considerable between‐study heterogeneity, researchers may decide to trim the default RoBMA ensemble by assigning prior probability zero to the fixed effects models, and consequently drawing conclusions from only the random effects models.

We have implemented RoBMA‐PSMA in a new version of the RoBMA R package.^61^ Also, for researchers with little programming expertise we will implement the methodology in the open‐source statistical software package JASP.^92^, ^93^ We hope that these publicly‐shared statistical packages will encourage researchers across different disciplines to adopt these new methods for accommodating potential publication bias and draw conclusions that are rich, robust, and reliable.

## AUTHOR CONTRIBUTIONS

All authors jointly generated the idea for the study. František Bartoš programmed the analysis, conducted the simulation study, and analyzed the data. František Bartoš and Maximilian Maier wrote the first draft of the manuscript and all authors critically edited it. All authors approved the final submitted version of the manuscript.

## CONFLICT OF INTEREST

František Bartoš declares that he owns a negligible amount of shares in semiconductor manufacturing companies that might benefit from a wider application of computationally intensive methods such as RoBMA‐PSMA. The authors declare that there were no other conflicts of interest with respect to the authorship or the publication of this article.

## Supporting information


**Appendix S1**. Supporting Information.

## Data Availability

The data and R scripts for performing the analyses and simulation study are openly available on OSF at https://osf.io/fgqpc/.

## Endnotes

* As measured by the geometric mean over researchers' self‐admission rate, prevalence estimate (the estimate for the percent of other researchers who had engaged in a behavior), and an admission estimate. For the admission estimates the number of people reporting to have engaged in a given QRP was divided by researchers estimate for the proportion of other researchers that would admit that they engaged in this QRP.

^†^
Both simple and inclusion Bayes factors are commonly denoted as BF since they are still Bayes factors and the same rules and interpretations apply to them.

^‡^
See^23^, ^58^, ^59^ for selection models based on effect sizes and standard errors.

^§^
We use the Fisher's *z* scale for model fitting because it makes standard errors and effect sizes independent under the model without publication bias. This is an important prerequisite to test for the presence/absence of publication bias with the PET‐PEESE models introduced later. The effect sizes and standard errors are transformed using the popular formulas for effect size transformations. See the appendix in Haaf and colleagues^62^ for proof that Fisher's *z* is a variance stabilizing transformation for Cohen's *d*. Prior distributions are linearly re‐scaled from Cohen's *d* to Fisher's *z*, in the same manner as in metaBMA R package.^63^

^**^
The reported lower bound of the credible interval of 0.000 is not a coincidence and will be encountered more often than in conventional frequentist methods. The 0.000 lower bound is a consequence of averaging posterior estimates across all models, including models that specify *μ* = 0 (see Equation (9)). When a notable proportion of the posterior model probabilities is accumulated by models assuming the absence of the effect, the model‐averaged posterior distribution for effect size will include a sizeable point mass at *μ* = 0. Consequently, the lower credible internal bound is then “shrunk” to 0.

^††^
This RRR category includes “replications published according to the ‘registered replication report’ format in the journals ‘Perspectives on Psychological Science’ and ‘Advances in Methods and Practices in Psychological Science’; and (2) The ‘Many Labs’ projects in psychology” (p. 424).^34^

^‡‡^
We also tried to estimate the remaining methods on the Fisher *z* scale; however, doing so reduced the performance of some of the other methods.

^§§^
This corresponds to the definition of the FPR and FNR indices from Kvarven and colleagues.^34^ However, it is important to note that a statistically non‐significant result is not generally a valid reason to “accept” the null hypothesis.^81^, ^82^

^***^
Note that this is not a general pattern and RoBMA often results in compelling evidence, either in favor of the absence or in favor of the presence of an effect.^83^, ^84^

^†††^
For the methods used in Hong & Reed,^27^ we recalculated the result based on a sample matching the 300 replications per conditions used for the RoBMA methods, using the per‐replication estimates shared by the authors.

^‡‡‡^
The QRPs simulated by Carter and colleagues^25^ results in a strongly positively skewed distribution of effect sizes. While RoBMA‐PSMA contains selection models with weight functions that well adjusts for publication bias simulated by Carter and colleagues,^25^ the additional skew generated by these QRPs results in misspecification of the best fitting models and consequent overcorrection of the meta‐analytic effect size. The simpler original RoBMA does not contain the appropriate one‐sided weight function. The skewed distribution of effect sizes does not introduce a strong systematic bias and it, as other methods, ironically result in better performance in the QRP environment of Carter and colleagues^25^ due to more layers of specific model misspecification. The remaining simulation environments produce bias in a more diverse manner and do not lead to such strongly skewed distributions of effect sizes. As a result, RoBMA‐PSMA does not generally suffer from systematic bias.

^§§§^
This transformation has been used to compare and rank top scientists across different criteria of scientific impact.^89^

^****^
Many of these methods either remove data (WAAP‐WLS) or impute data (trim‐and‐fill), which makes a comparison via marginal likelihood impossible.
