# Supplementary material for: Robust Bayesian meta‐analysis: Model‐averaging across complementary publication bias adjustment methods
Source: Res Synth Methods. 2022 Aug 7;14(1):99–116. doi: 10.1002/jrsm.1594 (PMC10087723; doi:10.1002/jrsm.1594)
Supplement: Supplementary file 1 — Appendix S1. Supporting Information. [file JRSM-14-99-s001.pdf]

Appendix A: Model Specifications

Most of the model specification is identical to the one described in Appendix A of Maier et al. (2022). The individual models employed in the ensemble (Table 1) differ in terms of the prior distribution specified over the effect size parameter (μ), the heterogeneity parameter (τ), and the way they adjust for publication bias (ω). If we group the models according to the way they adjust for publication bias, we can differentiate between the following model types based on the likelihood function.

Models Assuming No Publication Bias

Models assuming no publication bias use a normal likelihood to model the observed effect sizes y based on the observed standard errors se from K studies,

$$y_k \sim \text{Normal}(\mu, \tau^2 + se_k^2). \tag{1}$$

If the specific model assumes absence of an effect or heterogeneity, it further simplifies by setting $\mu = 0$ and $\tau = 0$. Otherwise, the corresponding prior distributions for μ and τ needs to be specified to obtain the complete model.

Models Adjusting for Publication Bias Based on The Relationship Between Standard Errors and Effect Sizes

Models correcting for publication bias by adjusting for the relationship between standard errors/variances and effect sizes use a normal likelihood as the models assuming no publication bias; however, they add a regression parameter that adjusts for the relationship between effect sizes and standard errors (PET) or the effect sizes and variances (PEESE),

$$\begin{aligned} y_k &\sim \text{Normal}(\mu + \text{PET} \times se_k, \tau^2 + se_k^2), \\ y_k &\sim \text{Normal}(\mu + \text{PEESE} \times se_k^2, \tau^2 + se_k^2). \end{aligned} \tag{2}$$

As before, in the case that the specific model assumes absence of the effect, or heterogeneity, it further simplifies by setting $\mu = 0$ and $\tau = 0$. Otherwise, the

corresponding prior distributions for μ, τ, and PET or PEESE needs to be specified to obtain the complete model.

Selection Models

Selection models use a weighted likelihood function to incorporate the publication probabilities, ω, into the likelihood function for the observed effect sizes,

$$y_k \sim \text{Weighted-normal}(\mu, \tau^2 + se_k^2, \omega). \tag{3}$$

Weighted-normal stands for a likelihood function of a weighted normal distribution, with mean μ, variance σ^2, weights ω, and a cumulative probability function of a standard normal distribution Φ, that is further differentiated accordingly whether the one-sided or two-sided selection is assumed,

$$\text{Weighted-normal}_{\text{one-sided}}(y \mid \mu, \sigma^2, \omega) = \frac{\text{Normal}(y \mid \mu, \sigma^2) \times w(\omega, p, c)}{\int \text{Normal}(x \mid \mu, \sigma^2) \times w(\omega, 1 - \Phi(x/\sigma), c)dx}, \tag{4}$$

$$\text{Weighted-normal}_{\text{two-sided}}(y \mid \mu, \sigma^2, \omega) = \frac{\text{Normal}(y \mid \mu, \sigma^2) \times w(\omega, p, c)}{\int \text{Normal}(x \mid \mu, \sigma^2) \times w(\omega, (1 - \Phi(|x/\sigma|)) \times 2, c)dx},$$

where the weights ω are assigned based on the one or two-sided p-values, p, and N cutoffs c through the weight function w,

$$w(\omega, p, c) = \begin{cases} \omega_1, & \text{if } p > c_1 \\ \omega_n, & \text{if } c_n < p \leq c_{n+1} \\ ... \\ 1, & \text{if } p \leq c_N \end{cases} \tag{5}$$

Again, in the case that the specific model assumes absence of the effect, or heterogeneity, it further simplifies by setting $\mu = 0$ and $\tau = 0$ respectively. Otherwise, the corresponding prior distributions for μ, τ, and ω needs to be specified to obtain the complete model.

Appendix B: Parameter Prior Distributions

Table 1 outlines the default prior distributions used throughout the manuscript. The specified default prior distributions can be viewed as a sensible starting options tested in

simulations. However, one of the advantages of Bayesian statistics is that it allows researchers to flexibly specify and test different hypotheses. We urge researchers to specify their own prior distributions directly corresponding to the hypotheses of interest. See Bartoš et al. (in press) for a tutorial where we explain how to specify different alternative and/or null hypotheses with RoBMA. Here, we outline the rationale for the default prior distributions used in this manuscript.

Effect Size (μ)

For the effect size μ, we use a standard normal, $\text{Normal}(0, 1)$, as the default prior distribution. We already used this distribution when introducing the previous version of the method (Maier et al., 2022). The standard normal prior distribution specifies a wide a range of plausible values for effect sizes, yet it has thinner tail than a frequently used $\text{Cauchy}(0, 1)$ prior distribution. The thinner tails of the standard normal distribution reduce the prior probability of very large effect sizes that we deem as less plausible in meta-analytic settings. Another choice for prior distribution for μ, used in the robustness analysis of Kvarven et al. (2020, Appendi C), might be $\text{Student-t}_{[0,\infty]}(0.35, 0.102, 3)$, so-called "Oosterwijk prior" (Gronau et al., 2020). The Oosterwijk prior is a shifted and scaled student-t distribution with location 0.35, scale 0.102, and three degrees of freedom, truncated to have mass only on positive effect sizes. We consider Oosterwijk prior to be a reasonable specification for effects that are known to be of small-to-medium size and it has been used in previous studies (e.g., Gronau et al., 2017; Landy et al., 2020).

Heterogeneity (τ)

For the heterogeneity τ, we use an inverse-gamma, $\text{InvGamma}(1, 0.15)$, as the default prior distribution. We also used this distribution in Maier et al. (2022) and it is based on heterogeneity estimates from meta-analyses in psychology recorded by van Erp et al. (2017). This prior distribution was used in previous studies (e.g., Gronau et al., 2017;

Landy et al., 2020) and it is also the default choice of the `metaBMA` R package (Heck et al., 2019).

Publication Bias Regression Coefficients For PET and PEESE

For the PET and PEESE regression coefficients, we use Cauchy, $\text{Cauchy}_{[0,\infty]}(0, 1)$ and $\text{Cauchy}_{[\infty]}(0, 5)$, as the default prior distributions. Equation 2 shows that the PET and PEESE regression coefficients can be thought as a bias of studies with a given standard error or variance. Since standard errors (and subsequently variances) are dependent on the sample size for standardized effect size measures, we derived the range of plausible values for the prior distribution based on the regression coefficients based on small sample studies ($\text{N} = 25, 50, 100$) and values of medium to large bias ($\text{bias} = 0.30, 0.40, 0.50$). The resulting values of PET regression coefficients are summarized in Table 1 and PEESE regression coefficients in Table 2. We conclude that the $\text{Cauchy}_{[0,\infty]}(0, 1)$ and $\text{Cauchy}_{[0,\infty]}(0, 5)$ prior distribution cover the range reasonably well and still allow for larger values in case that our initial assessment was incorrect.

Table 1

PET Regression Coefficients Based on Theoretical Sample Sizes and Degrees of Bias

Bias	0.30	0.40	0.50
N = 25	0.75	1.00	1.25
N = 50	1.06	1.41	1.77
N = 100	1.50	2.00	2.50

Table 2

PEESE Regression Coefficients Based on Theoretical Sample Sizes and Degrees of Bias

Bias	0.30	0.40	0.50
N = 25	1.88	2.50	3.13
N = 50	3.75	5.00	6.25
N = 100	7.50	10.00	12.50

To assess the robustness of our results in the Kvarven et al. (2020) example, we collected the estimated PET and PEESE regression coefficients from conditions assuming presence of the publication bias in the simulation study. We fitted gamma distributions to the simulation-based PET and PEESE regression coefficients using maximum likelihood and obtained $\text{Gamma}(2.84, 2.19)$ and $\text{Gamma}(2.32, 0.86)$ shape and rate parameterized prior distributions for PET and PEESE regression coefficients. Both of the prior distributions show a more concentrated prior probability density around 1.30 and 2.70 with a much thinner tail, making them more informed.
Notably, when transforming prior distributions for PET and PEESE regression coefficients to a different effect size scale, the prior distribution for the PET regression coefficient does not change – the scaling of the effect size corresponds to scaling of the standard error when using approximate linear transformation, however, the PEESE regression coefficient changes with the inverse of the approximate linear transformation applied to the effect size and standard errors.

Publication Bias Weights (ω)

For the publication bias weights ω, we use unit cumulative Dirichlet distributions, as the default prior distributions. In the case of a weight function with only one step, the unit cumulative Dirichlet distribution simplifies to a uniform distribution on interval from zero to one. In the more complex cases, the unit cumulative Dirichlet distributions assigns prior

probabilities across the possible weights, constraining them to be increasing, bound between zero and one, and allowing for variation in the predicted values.

Similarly to the prior distribution for the PET and PEESE regression coefficient, we assess the robustness of our results in the Kvarven et al. (2020) example by fitting cumulative Dirichlet distributions to the estimated publication bias weights based on the simulation study using maximum likelihood. Table 3 summarizes the simulation-based prior distribution and shows that the first parameter is usually larger resulting in a smaller step from significant to the non-significant studies, in other words, a more optimistic prediction regarding publication bias.

Table 3

Simulation-Based Prior Distribution for Publication Bias Weight Functions

Weight function	Prior distribution
$\omega_{\text{Two-sided}(.05)}$	$\text{CumDirichlet}(2.49, 0.83)$
$\omega_{\text{Two-sided}(.1,.05)}$	$\text{CumDirichlet}(2.88, 0.98, 0.99)$
$\omega_{\text{One-sided}(.05)}$	$\text{CumDirichlet}(2.61, 0.89)$
$\omega_{\text{One-sided}(.05,.025)}$	$\text{CumDirichlet}(2.92, 0.95, 0.75)$
$\omega_{\text{One-sided}(.5,.05)}$	$\text{CumDirichlet}(3.17, 0.80, 0.83)$
$\omega_{\text{One-sided}(.5,.05,.025)}$	$\text{CumDirichlet}(3.24, 1.02, 0.68, 0.66)$

Appendix C: Robustness of the Kvarven et al. (2020) Results Across Different Prior Specifications

To assess the robustness of the results on the empirical data sets provided by Kvarven et al. (2020), we repeated the analysis conducted in the "Evaluating RoBMA on Registered Replication Reports" section with different parameter prior distributions. First, we exchanged the default standard normal prior for the effect size μ with the Oosterwijk prior distribution, $\text{Student-t}_{[0,\infty]}(0.35, 0.102, 3)$. Second, we exchanged the default prior distributions for the PET and PEESE regression coefficients and the publication bias

weights ω for the simulation-based prior distributions. Finally, we exchanged the default prior distributions for both parameters simultaneously (a detailed description of the prior distributions can be found in Appendix B).

We found that all RoBMA models performed the best under the simulation-based prior distribution for the PET and PEESE regression coefficients and publication bias weights and the worst under the Oosterwijk prior distribution. We attribute the inferior performance of the Oosterwijk prior distribution to the fact that three replication studies resulted in negative estimates that are unattainable under the prior distribution restricted to positive numbers. However, even though there were some differences in how the RoBMA models performed under different prior distributions, the results were still in line with our previous conclusions.

Figure 1 compares the model-averaged posterior effect size estimate from RoBMA-PSMA with the default prior distribution specification (blue) to model-averaged posterior effect size estimates from RoBMA-PSMA with the above three alternative prior distributions for each of the 15 RRRs from Kvarven et al. (2020). In general, the figure shows consistent results across prior distributions; however, a closer look reveals that the informed Oosterwijk prior distribution pulls the model-averaged posterior for effect size towards its prior location (i.e., $\mu = 0.35$). Also note that under the Oosterwijk prior, the lower bounds of the credible interval do not cross zero – it is impossible to obtain a negative estimate when this has been ruled out a priori by restricting the prior range to the positive real line.

Table 4

Performance of RoBMA with Different Priors in the Kvarven et al. (2020) example.

Method	FPR	FNR	Undecided	OF	Bias	RMSE
Oosterwijk prior (μ)						
RoBMA-PSMA	0.286	0.000	0.667	1.446	0.073	0.204
RoBMA-old	0.714	0.000	0.133	2.050	0.172	0.224
Simulation-based prior						
RoBMA-PSMA	0.143	0.000	0.800	1.080	0.013	0.160
RoBMA-old	0.714	0.000	0133.	2.021	0.167	0.212
Oosterwijk prior (μ) & simulation-based prior						
RoBMA-PSMA	0.143	0.000	0.667	1.358	0.059	0.192
RoBMA-old	0.714	0.000	0.133	2.032	0.169	0.219

Note. FPR = false positive rate, FNR = false negative rate, Undecided = undecided evidence, OF = overestimation factor, and RMSE = root mean square error.

Appendix D: Robustness of the Kvarven et al. (2020) to the Selection of Registered Replication Reports

To further assess the robustness of the results on the empirical data sets provided by Kvarven et al. (2020), we repeated the analysis conducted in the "Evaluating RoBMA on Registered Replication Reports" section with a non-parametric bootstrap of the data set. We performed a 1000 repetitions, each of which sampled 15 RRR data sets with replacement which allowed us to assess the dependency of the reported results on the particular data.

Table 5 summarizes results from the non-parametric bootstrap as 95% quantile intervals. We found that the false positive and false negative rates estimates were highly variable since each of them was based only on a subset of the RRR (seven not statistically significant and eight statistically significant). Despite the added uncertainty depicted via

Figure 1

Robustness Check of Effect Size Estimates with 95% CIs Comparing RoBMA-PSMA with the Default Prior Distribution to Three Alternative Prior distribution Specifications for the 15 Experiments Included in Kvarven et al. (2020).

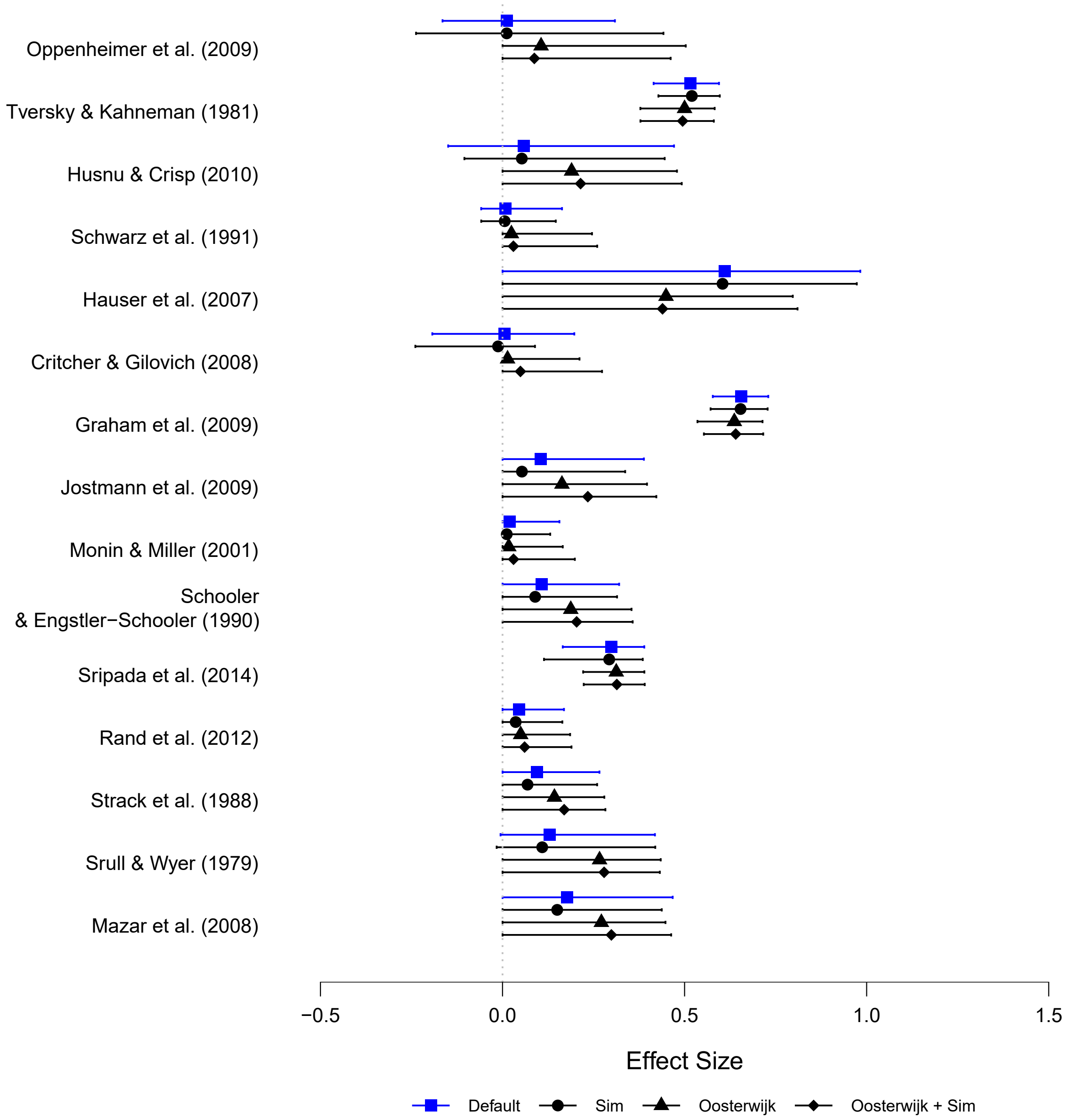

Note. Estimates are reported on the Cohen's d scale. "Sim" corresponds to simulation based priors for the publication bias adjustment part and "Oosterwijk" corresponds to an informed prior distribution expecting small-to-medium effect sizes (Student-$t_{[0,\infty]}(0.35, 0.102, 3)$).

the quantile intervals of overestimation, bias, and RMSE estimates, the results were aligned with our previous conclusions. Moreover, the higher quantile limit of bias and RMSE of the RoBMA-PSMA was around or bellow lower quantile limits of many other methods.

Appendix E: Performance Under the Absence of Publication Bias

In the "Evaluating RoBMA on Registered Replication Reports" section, all Registered Replication Reports (RRR) found lower effect size estimates than the original meta-analyses. To assess whether RoBMA's performance can be explained by a systematic underestimation of effect sizes, we estimated RoBMA and the remaining publication bias correction methods on data from Many Labs 2 (Klein et al., 2018). Many Labs 2 is a collection of different RRR attempting to replicate 28 classic and contemporary findings from psychology across multiple participating labs (N = 125). Since each finding was replicated by about half of the labs following the same RRR protocol, we can be certain about the absence of publication bias in the collection of the lab estimates. Consequently, we can establish the "Gold Standard" effect size estimate for each of the 28 psychological findings by applying a fixed-effect meta-analysis to the corresponding effect size estimates from the different labs (only one heterogeneity estimate τ was larger than 0.2). If RoBMA's previous performance was a result of systematic underestimation, we would expect to find significantly underestimated effect size estimates (the positive bias of the random-effect meta-analytic models in the "Evaluating RoBMA on Registered Replication Reports" section was 0.259).

The results are summarized in Table 6. We found that RoBMA-PSMA showed a small negative bias and slightly larger RMSE than most of the remaining methods. However, the increase in bias and RMSE in the Many Labs 2 data is decisively outweighed by the advantage in performance obtained in the "Evaluating RoBMA on Registered Replication Reports" section. This result, in conjunction with the results reported in the "Evaluating RoBMA Through Simulation Studies" section, show that the RoBMA's performance

Table 5

Robustness of Performance of 13 Publication Bias Correction Methods to Selection of the Kvarven et al. (2020) Test Set Comprised of 15 Meta-analyses and 15 Corresponding "Gold Standard" Registered Replication Reports (RRR).

Method	FPR / Undecided	FNR / Undecided	Overestimation	Bias	RMSE
RoBMA-PSMA	[0.000, 0.444] / [0.556, 1.000]	[0.000, 0.000] / [0.429, 1.000]	[0.744, 2.261]	[-0.054, 0.114]	[0.106, 0.214]
AK2	*[0.000, 0.000] / —*	*[0.000, 1.000] / —*	*[-0.758, 3.551]*	*[-0.326, 0.098]*	*[0.040, 0.453]*
PET-PEESE	[0.000, 0.429] / —	[0.143, 0.857] / —	[0.447, 2.343]	[-0.067, 0.176]	[0.108, 0.361]
EK	[0.000, 0.429] / —	[0.143, 0.857] / —	[0.527, 2.541]	[-0.063, 0.202]	[0.124, 0.403]
RoBMA-old	[0.333, 1.000] / [0.000, 0.667]	[0.000, 0.000] / [0.000, 0.000]	[1.417, 4.345]	[0.107, 0.237]	[0.162, 0.264]
3PSM	[0.333, 1.000] / —	[0.000, 0.400] / —	[1.462, 4.980]	[0.117, 0.271]	[0.177, 0.309]
4PSM	[0.363, 1.000] / —	[0.143, 0.833] / —	[1.025, 4.543]	[0.005, 0.243]	[0.209, 0.324]
TF	*[0.500, 1.000] / —*	*[0.000, 0.000] / —*	*[1.541, 5.319]*	*[0.127, 0.289]*	*[0.185, 0.325]*
AK1	[0.544, 1.000] / —	[0.000, 0.000] / —	[1.571, 5.378]	[0.145, 0.291]	[0.202, 0.320]
p-curve		/ —	[1.606, 5.350]	[0.134, 0.319]	[0.208, 0.362]
p-uniform	[0.000, 1.000] / —	[0.000, 0.750] / —	[1.587, 5.349]	[0.136, 0.316]	[0.210, 0.365]
WAAP-WLS	[0.544, 1.000] / —	[0.000, 0.400] / —	[1.675, 5.296]	[0.157, 0.330]	[0.186, 0.392]
RE	[1.000, 1.000] / —	[0.000, 0.000] / —	[1.686, 6.032]	[0.174, 0.342]	[0.225, 0.385]

Note. FPR / Undecided = false positive rate / undecided evidence under no effect, FNR/ Undecided = false negative rate/ undecided evidence under an effect, OF = overestimation factor, and RMSE = root mean square error. The table presents 95% bootstrapped percentile intervals from 1000 samples with results conditional on convergence. The results in *gray italic* are conditional on convergence: trim and fill did not converge in one case and AK2 did not converge in 10 cases.

cannot be explained by a systematic underestimation of effect sizes.

Table 6

Performance of 13 Publication Bias Correction Methods for 28 Meta-Analysis from Many Labs 2 Compared to a "Gold Standard" established with Fixed-Effect Meta-Analytic Models.

Method	FPR	FNR	Undecided	OF	Bias	RMSE
WAAP-WLS	0.000	0.056		1.005	0.002	0.011
TF	0.100	0.000		0.991	-0.004	0.044
Random Effects (DL)	0.000	0.000		1.035	0.013	0.035
3PSM	0.000	0.000		1.033	0.013	0.042
RoBMA-old	0.000	0.000	0.071	0.961	-0.015	0.043
AK1	0.000	0.056		1.083	0.017	0.044
4PSM	0.000	0.167		1.048	0.019	0.063
AK2	*0.167*	*0.200*	*0.000*	*0.631*	*0.020*	*0.051*
RoBMA-PSMA	0.000	0.000	0.214	0.897	-0.040	0.070
PET-PEESE	0.100	0.444		0.820	-0.070	0.170
p-curve				*1.352*	*0.058*	*0.195*
EK	0.100	0.444		0.733	-0.103	0.259
$p-$uniform	0.571	0.182		1.353	0.155	0.745

Note. FPR = false positive rate, FNR = false negative rate, Undecided = undecided evidence, OF = overestimation factor, and RMSE = root mean square error. The results in *gray italic* are conditional on convergence: p-uniform and p-curve did not converge in four cases (p-uniform also did not provide test for the effect in 10 cases) and AK2 did not converge in 17 cases. The rows are ordered based on combined log scores performance of the abs(log(OF), abs(Bias), and RMSE (not shown).

Appendix F: Additional Results from the Hong and Reed (2020) Simulation Study

Table 7

Mean Square Error (MSE) in the Carter et al. (2019) simulation environment stratified by publication bias.

Rank	No-QRP	MSE	Medium-QRP	MSE	High-QRP	MSE
1	*3PSM*	*0.012*	RoBMA-old	0.011	RoBMA-old	0.011
2	RoBMA-old	0.013	WAAP-WLS	0.018	WAAP-WLS	0.018
3	RoBMA-PSMA	0.014	*p*-uniform	0.021	*p*-uniform	0.019
4	WAAP-WLS	0.018	TF	0.023	TF	0.025
5	TF	0.018	*3PSM*	*0.023*	*p*-curve	0.029
6	*4PSM*	*0.021*	PET-PEESE	0.027	PET-PEESE	0.032
7	PET-PEESE	0.022	EK	0.033	*3PSM*	*0.035*
8	EK	0.027	*4PSM*	*0.033*	EK	0.038
9	Random Effects (DL)	0.039	*AK2**	*0.034*	*4PSM*	*0.040*
10	*p*-uniform	0.042	RoBMA-PSMA	0.039	Random Effects (DL)	0.052
11	*p*-curve	0.156	*p*-curve	0.041	RoBMA-PSMA	0.056
12	AK1*	0.620	Random Effects (DL)	0.047	AK1*	0.134
13	*AK2**	*2.515*	AK1*	0.086	*AK2**	*6.127*

Note. *The difference of performance in terms of MSE for AK1 and AK2 between our and Hong and Reed (2020) is a result of us not omitting 5% of the most extreme estimates.

Methods in *gray italic* converged in less than 90% repetitions in a given simulation environment.

Different columns correspond to the conditions described in Carter et al. (2019); "no-QRP" condition corresponds to lack of questionable research practices (QRPs), "medium-QRP" condition corresponds to mix of no QRPs (30%), moderate QRPs (50%), and strong QRPs (20%), and "high-QRP" condition corresponds to a mix of no QRPs (10%), moderate strategy QRPs (40%), and strong QRPs (50%).

Table 8

Bias in the Carter et al. (2019) simulation environment stratified by publication bias.

Rank	None	Bias	Medium	Bias	High	Bias
1	*3PSM*	*0.028*	PET-PEESE	0.059	WAAP-WLS	0.063
2	*4PSM*	*0.041*	WAAP-WLS	0.062	RoBMA-old	0.067
3	RoBMA-PSMA	0.045	RoBMA-old	0.063	PET-PEESE	0.070
4	PET-PEESE	0.047	AK1	0.065	AK1	0.071
5	EK	0.050	EK	0.075	EK	0.093
6	WAAP-WLS	0.061	*3PSM*	*0.091*	*p*-uniform	0.094
7	RoBMA-old	0.063	TF	0.094	*p*-curve	0.096
8	AK1	0.064	*p*-uniform	0.095	TF	0.100
9	*AK2*	*0.067*	*p*-curve	0.100	*3PSM*	*0.124*
10	TF	0.080	*4PSM*	*0.113*	*4PSM*	*0.134*
11	Random Effects (DL)	0.128	*AK2*	*0.123*	RoBMA-PSMA	0.161
12	*p*-uniform	0.129	RoBMA-PSMA	0.124	*AK2*	*0.166*
13	*p*-curve	0.158	Random Effects (DL)	0.154	Random Effects (DL)	0.167

Note. Methods in *gray italic* converged in less than 90% repetitions in a given simulation environment.

Different columns correspond to the conditions described in Carter et al. (2019); “no-QRP” condition corresponds to lack of questionable research practices (QRPs), “medium-QRP” condition corresponds to mix of no QRPs (30%), moderate QRPs (50%), and strong QRPs (20%), and “high-QRP” condition corresponds to a mix of no QRPs (10%), moderate strategy QRPs (40%), and strong QRPs (50%).

References

Bartoš, F., Maier, M., Quintana, D., & Wagenmakers, E.-J. (in press). Adjusting for publication bias in JASP and R — Selection models, PET-PEESE, and robust Bayesian meta-analysis. *Advances in Methods and Practices in Psychological Science*. https://doi.org/10.31234/osf.io/75bqn

Carter, E. C., Schönbrodt, F. D., Gervais, W. M., & Hilgard, J. (2019). Correcting for bias in psychology: A comparison of meta-analytic methods. *Advances in Methods and Practices in Psychological Science*, *2*(2), 115–144. https://doi.org/10.1177/2515245919847196

Gronau, Q. F., Ly, A., & Wagenmakers, E.-J. (2020). Informed Bayesian t-tests. *The American Statistician*, *74*(2), 137–143. https://doi.org/10.1080/00031305.2018.1562983

Gronau, Q. F., van Erp, S., Heck, D. W., Cesario, J., Jonas, K. J., & Wagenmakers, E.-J. (2017). A Bayesian model-averaged meta-analysis of the power pose effect with informed and default priors: The case of felt power. *Comprehensive Results in Social Psychology*, *2*(1), 123–138. https://doi.org/10.1080/23743603.2017.1326760

Heck, W., D., Gronau, F., Q., Wagenmakers, & E.-J. (2019). *MetaBMA: Bayesian model averaging for random and fixed effects meta-analysis.* https://CRAN.R-project.org/package=metaBMA

Hong, S., & Reed, W. R. (2020). Using Monte Carlo experiments to select meta-analytic estimators. *Research Synthesis Methods*, *12*, 192–215. https://doi.org/10.1002/jrsm.1467

Klein, R. A., Vianello, M., Hasselman, F., Adams, B. G., Adams Jr, R. B., Alper, S., Aveyard, M., Axt, J. R., Babalola, M. T., Bahník, Š., et al. (2018). Many Labs 2: Investigating variation in replicability across samples and settings. *Advances in Methods and Practices in Psychological Science*, *1*(4), 443–490. https://doi.org/10.1177/515245918810225

Kvarven, A., Strømland, E., & Johannesson, M. (2020). Comparing meta-analyses and preregistered multiple-laboratory replication projects. *Nature Human Behaviour*, *4*(4), 423–434. https://doi.org/10.1038/s41562-019-0787-z

Landy, J. F., Jia, M. L., Ding, I. L., Viganola, D., Tierney, W., Dreber, A., Johannesson, M., Pfeiffer, T., Ebersole, C. R., Gronau, Q. F., et al. (2020). Crowdsourcing hypothesis tests: Making transparent how design choices shape research results. *Psychological Bulletin*, *146*, 451–479. https://doi.org/10.1037/bul0000308

Maier, M., Bartoš, F., & Wagenmakers, E.-J. (2022). Robust Bayesian meta-analysis: Addressing publication bias with model-averaging. *Psychological Methods*. https://doi.org/10.1037/met0000405

van Erp, S., Verhagen, J., Grasman, R. P., & Wagenmakers, E.-J. (2017). Estimates of between-study heterogeneity for 705 meta-analyses reported in Psychological Bulletin from 1990–2013. *Journal of Open Psychology Data*, *5*(1), Article 4. http://doi.org/10.5334/jopd.33
